# Unleashing the power within short-read RNA-seq for plant research: Beyond differential expression analysis and toward regulomics

**DOI:** 10.3389/fpls.2022.1038109

**Published:** 2022-12-08

**Authors:** Min Tu, Jian Zeng, Juntao Zhang, Guozhi Fan, Guangsen Song

**Affiliations:** ^1^ School of Chemical and Environmental Engineering, Wuhan Polytechnic University, Wuhan, China; ^2^ Guangdong Provincial Key Laboratory of Utilization and Conservation of Food and Medicinal Resources in Northern Region, Shaoguan University, Shaoguan, Guangdong, China

**Keywords:** plant transcriptomics, RNA-seq data analysis, alternative splicing, alternative polyadenylation, coexpression network, gene regulatory network, regulomics

## Abstract

RNA-seq has become a state-of-the-art technique for transcriptomic studies. Advances in both RNA-seq techniques and the corresponding analysis tools and pipelines have unprecedently shaped our understanding in almost every aspects of plant sciences. Notably, the integration of huge amount of RNA-seq with other omic data sets in the model plants and major crop species have facilitated plant regulomics, while the RNA-seq analysis has still been primarily used for differential expression analysis in many less-studied plant species. To unleash the analytical power of RNA-seq in plant species, especially less-studied species and biomass crops, we summarize recent achievements of RNA-seq analysis in the major plant species and representative tools in the four types of application: (1) transcriptome assembly, (2) construction of expression atlas, (3) network analysis, and (4) structural alteration. We emphasize the importance of expression atlas, coexpression networks and predictions of gene regulatory relationships in moving plant transcriptomes toward regulomics, an omic view of genome-wide transcription regulation. We highlight what can be achieved in plant research with RNA-seq by introducing a list of representative RNA-seq analysis tools and resources that are developed for certain minor species or suitable for the analysis without species limitation. In summary, we provide an updated digest on RNA-seq tools, resources and the diverse applications for plant research, and our perspective on the power and challenges of short-read RNA-seq analysis from a regulomic point view. A full utilization of these fruitful RNA-seq resources will promote plant omic research to a higher level, especially in those less studied species.

## Introduction

RNA-seq and its-derived techniques have been commercially available and routinely used by biological scientists, largely owing to the rapidly increased outputs of major sequencing platforms, improved sequencing accuracy and ever reduced costs (Stark et al., 2019). RNA-seq has shaped nearly every aspects of our understanding in plant research, from plant development and phytohorome signaling to plant metabolism and stress tolerance.

RNA-seq can be divided into the short-read (Nagalakshmi et al., 2008) and long-read RNA-seq technologies (Sharon et al., 2013). In short-read RNA-seq, Illumina sequencing platform has been dominant, while other platforms, such as Thermo Scientific platforms (e.g., Ion PGM and Ion S5) or the BGI Genomics platforms (e.g., DNBSEQ), have been frequently used in certain circumstances or been gaining attentions recently (Patterson et al., 2019; Foox et al., 2021). A short-read RNA-seq library is typically sequenced to a read depth of 10~30 million reads per sample with a read length varied from 50 to 200 bp. By contrast, a number of approaches (*e.g.*, Pacific Bioscience, PacBio and Oxford Nanopore, ONT) provide long, uninterrupted sequencing of a single RNA or DNA molecules, constituting the third generation of real-time fluorescence sequencing paradigm (Sharon et al., 2013; Cartolano et al., 2016; Oikonomopoulos et al., 2016). A typical long-read RNA-seq produces 500,000 to 10 million reads per run with a read length ranging from 1,000 to 50,000 bp depending on the technologies and platforms (Stark et al., 2019). The long-read sequencing platforms are particularly suited for *de novo* transcriptome assembly and identification of novel transcripts and isoforms, as these approaches overcome some intrinsic issues related to short-read sequencing.

While the rise of the long-read RNA-seq, the short-read RNA-seq still is dominating the current utilizations in plant sciences and has provided the majority of the data sets deposited in public sequencing databases. With the recent advancement of tools developed for analyzing short-read sequencing data, the RNA-seq technology can be used for various applications, including but not limited to: (1) *de novo* assembly of transcriptome with or without a reference genome; (2) detection of new transcripts or correction of existing gene structures based on RNA-seq evidence; (3) to obtaining the expression profiles at gene or transcript levels and to construct the expression atlas covering a range of conditions and tissue types; (4) to identify alternative splicing and alternative 5’ or 3’ untranslated regions (5’UTR or 3’UTR, respectively); (5) to construct gene co-expression networks (GCNs) and predict gene regulatory relationships in a large scale (also known as gene regulatory networks, GRN). Here, GCN stand for a network that can be constructed from a large set of RNA-seq data and includes multiple clusters or modules. The module represents a group of genes determined statistically with high correlation in their expression profiles and usually associations in their functions (reviewed in Gupta and Pereira, 2019). Notably, many genes within the same module do not represent the direct targets of their upstream regulators. Thus, to further disentangle the direct regulator-targets pairs from the indirectly regulated or co-expressed genes, prediction of GRNs is another important task in RNA-seq data analysis. Identification of GRNs can be achieved by harnessing the following resources: (1) identifying transcription factors (TFs) from co-expressed modules; (2) identifying a group of co-expressed genes with the statistically enriched cis-regulatory elements from a certain family of TF; (3) leveraging the information of direct TF targets by using existing results from chromatin immunoprecipitation sequencing (ChIP-seq) or DNA affinity purification sequencing (DAP-seq) experiments (O’Malley et al., 2016; Galli et al., 2020); (4) applying the well-established algorithms for GRN inference. While the many utilizations of RNA-seq, the differential gene expression (DGE) is still the most often used analysis in many plant researches, especially those carried on in crop species.

Here, we highlight typical examples of the tools and applications that have been used in the model plants (Arabidopsis and rice) and other major crops (*e.g.*, tomato, wheat, maize and soybean) (**Table 1**). These applications demonstrate the power and comprehensiveness of short-read, bulk RNA-seq analyses. Meanwhile, it is worth noting that DGE has long been the primary analysis in the RNA-seq studies of other less-studied plant species. In fact, many species, especially those minor crops, biomass crops or orphan crops, are key to provide sustainable agriculture and to reach global food and energy security. Particularly, major biomass crops, such as sorghum, sugarcane, *Miscanthus*, and switchgrass, have large yield of biomass and stress tolerance (Mullet et al., 2014; Boyles et al., 2019), justifying the significance for researching on gene expression and regulation associated with biomass composition and production.

**Table 1:** Summary of the representative resources and tools for analyzing the short-read RNA-seq data in plants.

Name	Reference	URL	Implementation	Classification^1^
Plant Reactome	Nathani et al., 2017 & Nathani et al., 2020	http://plantreactome.gramene.org	Web Page	Annotation
Strawberry	Liu and Dickerson, 2017	https://github.com/ruolin/strawberry	Stand Alone	Annotation
iDEP	Ge et al., 2018	http://ge-lab.org/idep/	R Package	Annotation
TransFlow	Seoane et al., 2018	https://github.com/seoanezonjic/TransFlow.	Stand Alone	Annotation
MorphDB	Zwaenepoel et al., 2018	http://bioinformatics.psb.ugent.be/webtools/morphdb/morphDB/index/.	Web Page	Annotation
PISO	Feng et al., 2019	http://cbi.hzau.edu.cn/piso/.	Web Page	Annotation
MapMan 4/Mercator4	Schwacke et al., 2019	https://www.plabipd.de/portal/legacy-mercator4	Web Page	Annotation
PlantCircBase	Chu et al.,2018	http://ibi.zju.edu.cn/plantcircbase/	Web Page	Annotation & Expr.
Gramene	Tello-Ruiz et al., 2018	http://www.gramene.org	Web Page	Annotation & Expr.
LeGOO	Carrere et al., 2020	https://www.legoo.org	Web Page	Annotation & Expr.
ZEAMAP	Gui et al., 2020	http://www.zeamap.com	Web Page	Annotation & Expr.
BarleyNet	Lee et al., 2020	http://www.inetbio.org/barleynet	Web Page	Annotation & Expr.
SAT-Assembler	Zhang et al., 2014	https://sourceforge.net/projects/sat-assembler/	Stand Alone	Assembler
BinPacker	Liu et al., 2016	http://sourceforge.net/projects/transcriptomeassembly/files/BinPacker_1.0.tar.gz/download	Stand Alone	Assembler
Rascaf	Song et al., 2016	https://github.com/mourisl/Rascaf.	Stand Alone	Assembler
IGB	Freese et al., 2016	http://bioviz.org/igb.	Web Page	Browser
eFP-Seq Browser	Sullivan et al., 2019	https://bar.utoronto.ca/eFP-Seq_Browser/	Web Page	Browser
RNAprof	Tran et al., 2016	http://rna.igmors.u-psud.fr/Software/rnaprof.php	Stand Alone	AS/APA
Apatrap	Ye et al., 2018	https://apatrap.sourceforge.io.	Stand Alone	AS/APA
**Name**	**Citation**	**URL**	**Implementation**	**Classification**
priUTR	Tu and Li, 2020	https://github.com/mint1234/3UTR-	Stand Alone	AS/APA
3D RNA-Seq	Guo et al., 2021	https://ics.hutton.ac.uk/3drnaseq	R Package	AS/APA
TEtranscripts	Jin et al., 2015	http://hammelllab.labsites.cshl.edu/software	Stand Alone	Expression
expVIP	Borrill et al., 2016	www.wheat-expression.com	Web Page	Expression
OryzaExpress	Kudo et al., 2017	http://plantomics.mind.meiji.ac.jp/OryzaExpress/	Web Page	Expression
BAR	Waese and Provart, 2016	http://bar.utoronto.ca	Web Page	Expression
DPMIND	Fei et al.2018	http://202.195.246.60/DPMIND/	Web Page	Expression
PEATmoss	Fernandez-Pizo et al., 2020	https://peatmoss.online.uni-marburg.de	Web Page	Expression
ASmir	Wang et al., 2019	http://forestry.fafu.edu.cn/bioinfor/db/ASmiR	Web Page	Expression
Soybean Expression Atlas	Machado et al.,2020	http://venanciogroup.uenf.br/resources/	Web Page	Expression
Grape-RNA	Wang et al., 2020	http://www.grapeworld.cn/gt/2	Web Page	Expression
CORNET	Van Bel and Coppens, 2017	http://bioinformatics.psb.ugent.be/cornet/	Web Page	Expr. & Coexp.
NaDH	Brockmoller et al., 2017	http://nadh.ice.mpg.de/	Web Page	Expr. & Coexp.
NorWood	Jokipii-Lukkari et al., 2017	http://norwood.congenie.org	Web Page	Expr. & Coexp.
AspWood	Sundell et al., 2017	http://aspwood.popgenie.org	Web Page	Expr. & Coexp.
RED	Xia et al., 2017	http://expression.ic4r.org	Web Page	Expr. & Coexp.
EXPath	Zheng et al.2017	http://expathtool.itps.ncku.edu.tw/	Web Page	Expr. & Coexp.
TomExpress	Zouine et al., 2017	http://tomexpress.toulouse.inra.fr	Web Page	Expr. & Coexp.
Maize eFP Brower	Hoopes et al., 2019	bar.utoronto.ca/efp_maize	Web Page	Expr. & Coexp.
ATTED	Obayashi et al., 2018	http://atted.jp	Web Page	Expr. & Coexp.
MCENet	Tian et al., 2018	http://bioinformatics.cau.edu.cn/MCENet/	Web Page	Expr. & Coexp.
AppleMDO	Da et al., 2019	http://bioinformatics.cau.edu.cn/AppleMDO/	Web Page	Expr. & Coexp.
**Name**	**Citation**	**URL**	**Implementation**	**Classification**
Melonet-DB	Yano et al., 2018	http://melonet-db.agbi.tsukuba.ac.jp/	Web Page	Expr. & Coexp. & Anno.
TPIA	Xia et al., 2019	http://tpia.teaplant.org	Web Page	Expr. & Coexp. & Anno.
Plant Regulomics	Ran et al., 2020	http://bioinfo.sibs.ac.cn/plant-regulomics.	Web Page	Expr. & Coexp. & Anno.
CSI	Penfold et al., 2015a & Penfold et al., 2015b	http://go.warwick.ac.uk/systemsbiology/software.	Stand Alone	Network construction
RSAT-Plants	Contreras-Moreira et al., 2016	http://plants.rsat.eu	Web Page	Network construction
tcgsaseq	Agniel and Hejblum, 2017	https://cran.r-project.org/web/packages/tcgsaseq.	R Package	Network construction
SeqEnrich	Becker et al., 2017	http://www.belmontelab.com	Stand Alone	Network construction
ExRANGES	Desai et al., 2017	http://github.com/DohertyLab/ExRANGES	R Package	Network construction
LSTrAP	Proost et al., 2017	https://github.molgen.mpg.de/proost/LSTrAP	Stand Alone	Network construction
RSAT	Nguyen et al., 2018	http://www.rsat.eu/	Stand Alone	Network construction
NetMiner	Yu et al., 2018	https://github.com/czllab/NetMiner.	Stand Alone	Network construction
ExpressWeb	Savelli et al., 2019	http://polebio.lrsv.upstlse.fr/ExpressWeb/	R Package	Network construction
HTRgene	Ahn et al., 2019	http://biohealth.snu.ac.kr/software/HTRgene.	R Package	Network construction
Compare Transcriptome Analysis	Lee et al., 2019	https://github.com/LiLabAtVT/CompareTranscriptome.git).	R Package	Network construction
JASPAR	Fornes et al., 2020	http://jaspar.genereg.net	Web Page	Network construction
GENIE3	Harrington et al., 2020	https://github.com/Uauy-Lab/GENIE3_scripts/	Stand Alone	Network construction
LSTrAP-Cloud	Tan et al., 2020	https://github.com/tqiaowen/LSTrAP-Cloud	Stand Alone	Network construction
RSAT	Ksouri et al., 2021	https://github.com/RSAT-doc/motif_discovery_clusters	Web Page	Network construction

**1.** The RNA-seq resources and tools are classified by their functions, including annotation, expression atlas (expression, or abbreviated as ‘Expr.’), co-expression analysis (abbreviated as ‘Coexp.’), alternative splicing and alternative polyadenylation (abbreviated as ‘AS/APA’), and network construction (tools for calculating coexpression networks or gene regulatory networks). These resources and tools are first sorted by classification and then by publication years.

**Figure 1:**
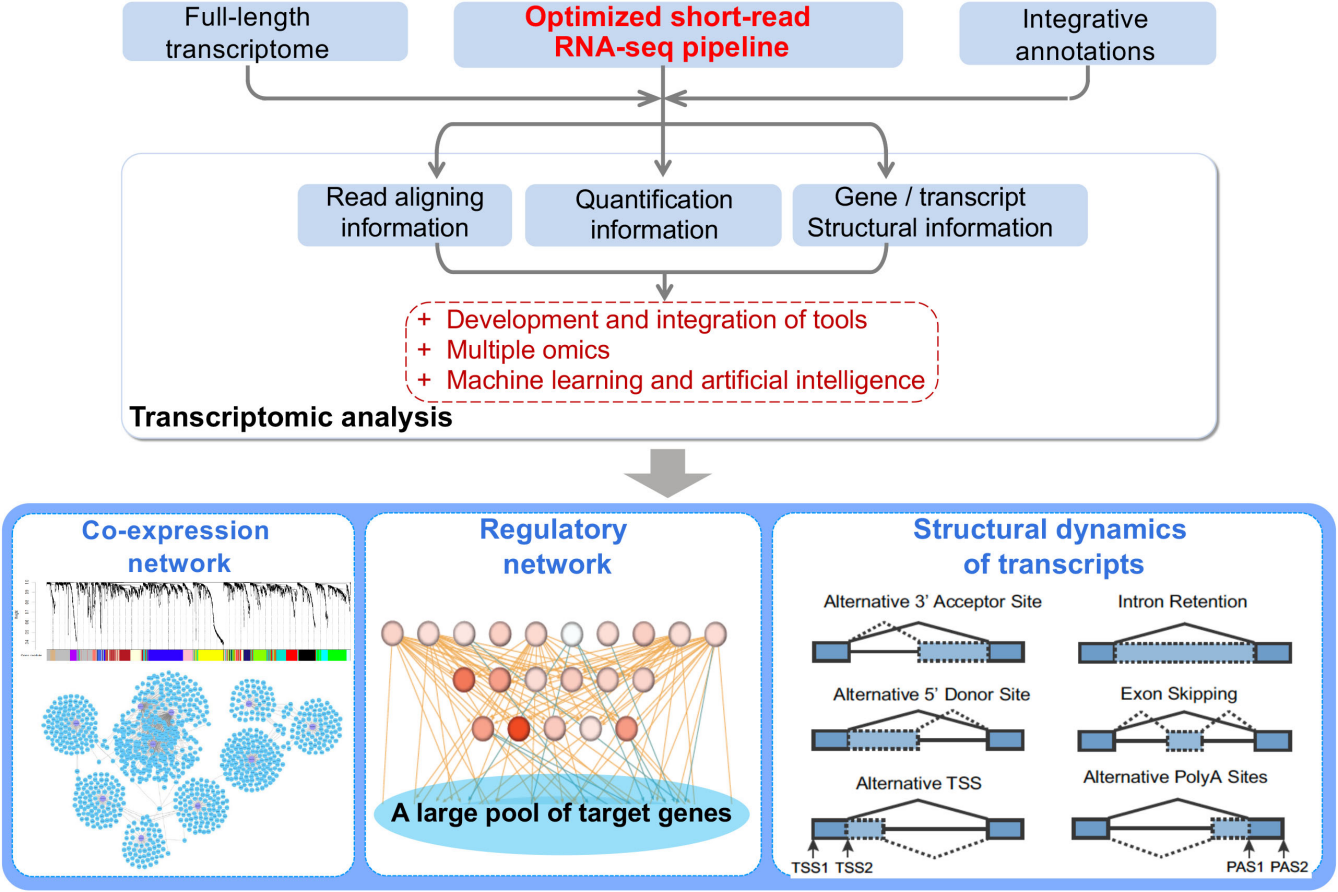
The power of short-read, bulk RNA-seq can be unleashed by integrating the following resources and tools related to RNA-seq analysis: **(1)** Full-length transcriptome can be achieved by full-length cDNA sequencing, PacBio Iso-seq or the Oxford Nanopore sequencing technologies, and these full-length transcriptomes can help to better annotate gene structures and serve as the basis for expression profiling at the transcript-level. **(2)** For many less-studied plant species, multiple functional annotation resources can be applied to provide a comprehensive annotation, facilitating biological interpretation of sets of DEG or gene networks. **(3)** Through application of the tools introduced here and in previous reviews, high-quality GCNs and GRNs can be made to prioritize hub genes or key regulators involved in the certain biological process or phenotypes.

The limited utilization of RNA-seq in the minor plant species has been partly due to: (1) the limited genomic resources; (2) lacking bioinformatic tools that are user friendly, with a graphical user interface, or well adapted to the omics data of various species. In this context, we summarize a variety of bioinformatic tools covering the diverse applications of bulk RNA-seq analysis to facilitate the full use of short-read RNA-seq data, and to help unleash the power of bulk RNA-seq in studies of plants, especially in the minor and under-utilized crops (**Table 1**; **Figure 1**). Notably, there have been several excellent reviews regarding the development of RNA-seq technologies, comprehensive summary of RNA-seq tools and calculation of GCNs and GRNs in plant sciences (Van Verk et al., 2013; Conesa et al., 2016; Proost and Mutwil, 2016; Gaudinier and Brady, 2016; Sahraeian et al., 2017; Saelens et al., 2018; Haque et al., 2018; Stark et al., 2019; Gupta and Pereira, 2019). We aim at neither comprehensively cataloguing the RNA-seq analysis tools for plant research, nor summarizing the achievements that RNA-seq have been reached in plant research. We emphasize that recent advancements in RNA-seq analysis tools allow to fully unleash the power of short-read, bulk RNA-seq in many plant species like biomass crops, to provide deep insights into gene regulation at multiple levels and to go toward regulomics, an analogous term to other omics that portraits transcription control in a genome-wide manner (Werner, 2003; Werner, 2004). Particularly, regulomics refers to the omic-scale study of gene expression regulation happened at transcriptional or post-transcriptional levels (Werner, 2004), such as the regulation between transcription factors/coregulators and their targets and the interaction between non-coding RNAs (e.g., miRNAs anf lncRNAs) and mRNAs.

## The applications of the short-read, bulk RNA-seq in plant sciences

The short-read RNA-seq technique includes several core steps, from RNA extraction, cDNA synthesis, adapter ligation, PCR amplification, to the sequencing of library and data analysis. Four key stages are required for the RNA-seq data analysis: (1) The first stage takes the raw sequencing reads to quality control and maps the quality-controlled reads to the transcriptome, which can be obtained from a reference genome or be assembled from transcriptomic data; (2) The second stage quantifies the number of reads mapped to each gene or transcript, producing an expression matrix; (3) The third stage modifies the expression matrix by normalization between samples, accounting for technical differences, and removing lowly expressed genes/transcripts; (4) The last stage calculates differentially expressed genes or transcripts by statistical models. Particularly, the number of computational tools for analyzing RNA-seq data has been increased dramatically in the recent decade (Stark et al., 2019). As such, substantial influences can be generated on the biological conclusions drawn from the RNA-seq data due to several aspects: differences in the computational approaches used, software parameters or statistical models selected and distinct combinations of the tools in a pipeline (Conesa et al., 2016). The optimal set of computational approaches for RNA-seq depends on the experimental setup, the biological questions being addressed and other factors, and is beyond the scope of our mini-review (Conesa et al., 2016; Sahraeian et al., 2017). However, several sets of RNA-seq tools are well recognized, representing the classic pipelines (Trapnell et al., 2012; Grabherr et al., 2012; Pertea et al., 2017). These includes five main components: (1) the splice-aware aligners (*e.g.*, TopHat, STAR, HISAT and HISAT 2; Kim et al., 2019) to map RNA-seq reads to the reference genome; (2) the tools for reads extraction [*e.g.*, HTSeq (Anders et al., 2014) and featureCount (Liao et al., 2014)]; (3) the tools for transcript construction (e.g., CuffLinks, StringTie) (Trapnell et al., 2012; Pertea et al., 2017); (4) the tools for estimates gene/transcript abundance [e.g., CuffDiff2, Ballgown and RSEM (Li and Dewey, 2011)]; and (5) the tools to identify differentially expressed genes or transcripts based on statistical analyses (such as edgeR (Robinson et al., 2010), DESeq2 (Love et al., 2014), Ballgown and CuffDiff2). The majority of the applications and computational tools summarized in the follow are compatible with these classic RNA-seq pipelines.

### RNA-seq data enhance transcriptome assembly

The number of plant species with at least one reference genome have multiplied dramatically over the past few years, with 798 land plant species having genome assemblies (as of Jan. 2021) (Marks et al., 2021). While these genomic resources greatly ease the RNA-seq analysis, still the complexity in plant genomes and transcriptomes presents major challenges in RNA-seq analysis. Many plant species feature large genomes (for example, the median sizes of currently sequenced monocots and eudicots respectively are more than 500 Mb) or complex auto- or allo- polyploid genomes with some hybridization and introgressions (Zhang et al., 2018; Zhao et al., 2021; Sun et al., 2022). Many genomes are expanded by repetitive sequences (such as transposons), making it difficult to achieve complete and accurate annotation of multi-exonic genes. Besides, alternative splicing (AS) and alternative polyadenylation (APA) further enhance transcriptome complexity. In addition, gene families commonly seen in the plant genomes are shaped by whole genome duplication, segmental duplication and tandem duplication. The members within a gene family or the homo-/homoeo-logous alleles (in polyploid) usually share high sequence similarity between each other, thus posing ad-ditional challenges in accurate quantification of the expression levels by using RNA-seq data.

To overcome these challenges, two strategies have been evolved when a reference genome is available: **(1)** to assembly transcripts first and then to quantify expression; **(2)** to simultaneously construct transcripts and to quantify expression. For the genome-guided transcriptome analysis, multiple pipelines have been established that differ in the algorithms used and the speed and computational resources required, including the classic TopHat-Cufflink-Cuffdiff pipeline (Trapnell et al., 2012) and HISAT-StringTie-Ballgown pipeline (Pertea et al., 2017), as well as the new “Strawberry” tool (Liu and Dickerson, 2017). By contrast, when a reference genome and gene annotations do not exist, a transcriptome needs to be firstly *de novo* assembled to facilitate expression quantification. However, *de novo* assembly based on short-read RNA-seq data usually leads to fractured and incomplete view of transcriptome, complicating downstream analysis (Malik et al., 2018). Several tools for *de novo* assembling full-length transcripts have become popular with different algorithms and features, such as Trinity (Haas et al., 2013), Oasis (Schulz et al., 2012), Trans-AbySS (Robertson et al., 2010), SOAPdenovo-Trans (Xie et al., 2014), Corset (Garber et al., 2011) and BinPacker (Liu et al., 2016). More recently, Grouper provides a complete pipeline for processing *de novo* transcriptomic analysis by using a new method for clustering assembled contigs (Malik et al., 2018). TransFlow provides a versatile workflow to enhance *de novo* transcriptome analyses and to annotate transcript structures more accurately by combining short-read and long-read sequencing data (Seoane et al., 2018).

### RNA-seq data empower the construction of expression atlas

Rapid accumulation of immense sets of RNA-seq data allows the establishment of expression atlantes. An expression atlas collects a large number of RNA-seq data from a certain species and re-analyzes these data using standardized, open-source pipelines to remove potential batch effects and any influences caused by other factors, such as different research groups, sequencing platforms and experiments (Papatheodorou et al., 2018). Establishing expression atlas has been proved very valuable in model organisms to promote not only omics studies but more importantly our understanding in gene functions, as clues to gene function can often be inferred by examining when and where a gene is expressed in the organism (Alberts et al., 2002). In model plants and major crops, such expression atlantes have served as key resources to the research community. For example, the information hub of Arabidopsis (TAIR; Berardini et al., 2015) and maize (MaizeGDB; Lawrence et al., 2008) have implemented with the expression atlas for each species. Maize expression atlas websites have been updated or built separately by multiple groups to integrate more RNA-seq data, other omics data sets or visualizations (Sekhon et al., 2013; Stelpflug et al., 2015; Tian et al., 2018; Hoopes et al., 2019; Gui et al., 2020). Similarly, the rice expression atlas has been updated from microarray to RNA-seq data sets and established by several groups respectively (Sato et al., 2013; Kudo et al., 2017; Xia et al., 2017). Recently, the expression atlantes have also been built for other important crops, such as tomato (TomExpress, Zouine et al., 2017), soybean (Machado et al., 2020), wheat (Borrill et al., 2016), barley (BarleyNet, Lee et al., 2020) and sorghum (Makita et al., 2015). The trend of building RNA-seq-based expression atlas has been spread to many less-studied plant species, for example, *Picea abies* (the Norwood database, Jokipii-Lukkari et al., 2017), *Populus tremula* (the Aspwood database, Sundell et al., 2017), chickpea (Kudapa et al., 2018), *Physcomitrella Paten* (Perroud et al., 2018; Fernandez-Pizo et al., 2020), tabacco (NaDH- Brockmoller et al., 2017), water melon (Melonet-DB - Yano et al., 2018), apple (AppleMDO- Da et al., 2019), tea (TPIA - Xia et al., 2019), grape (Wang et al., 2020), and *Medicago truncatula* (LeGOO- Carrere et al., 2020).

Notably, two types of the integrative websites are particularly valuable in facilitating comparative functional genomics and molecular breeding. (1) The expression atlas website includes a number of useful functions, from the visualization, comparison and functional enrichment of the omics data to comprehensive annotations of genes or gene families and useful functions such as primer design, BLAST and ortholog identification. (2) The RNA-seq data are further utilized to construct co-expression modules and integrated with other types of omics data, for example epigenomic data sets. In addition, major plant genomics websites (for instance, the Phytozome (Goodstein et al., 2011) Ensembl Plants (Bolster et al., 2017), and Gramene (Tello-Ruiz et al., 2018)) serve as the central data hub to link numerous plant genomes to those of the model species, which are well characterized and annotated. These iconic plant genomic hubs lay a solid foundation for transferring and comparing the omic information from model plants to less-studied species.

### RNA-seq data capture large-scale co-expression networks

One major cornerstone of the data-driven biological interpretation of large-scale RNA-seq data is to transform expression data into networks and modules. Among the network representation methods, co-expression network is the one that has been widely applied and successful in many species (Farber and Lusis, 2008). In a co-expression network, genes are connected by edges that quantify the similarity between gene expression patterns, and the genes expressed similarly are grouped together forming a co-expression module. Co-expression network can be calculated by different approaches, from correlation-based methods like Pearson Correlation Coefficiency (PCC) (D’haeseleer et al., 2000) and weighted gene co-expression network analysis (WGCNA) (Langfelder and Horvath, 2008; Langfelder and Horvath, 2012), to linear modelling (Vasilevski et al., 2012) and mutual information methods (Daub et al., 2004). Through the “guilt-by-association” principle, genes in a co-expression module possibly indicate similar functions and modes of transcriptional regulation (Wolfe et al., 2005), or similar cellular compartments of the protein products (Ryngajllo et al., 2011).

Over the past decade, high-quality co-expression networks and their hosting data hubs have served as a valuable resource to facilitate the gene functional studies in model plant species and many major crops, including Arabidopsis (Van Bel et al., 2017; Obayashi et al., 2018), rice (Xia et al., 2017), maize (Miao et al., 2017; Tian et al., 2018; Hoopes et al., 2019), and tomato (Zouine et al., 2017). More recently, co-expression networks have been built in other plant species (Kudapa et al., 2018), including some forest species with biomass purposes (Jokipii-Lukkari et al., 2017; Sundell et al., 2017), demonstrating the power of network representation in providing molecular functional insights into biomass production. Nonetheless, the biologists who work on less-studied plant species might neither have the bioinformatic skills nor afford the computational resources that are required to integrate large-scale RNA-seq data sets and to construct high-quality networks. Thus, user-friendly online or offline tools have been developed to lower the bar for co-expression-based analysis, such as the Kallisto-based LSTrAP pipeline (Proost et al., 2017), the LSTrAP-Cloud (Tan et al., 2020) and the ExpressWeb (Savelli et al., 2019). Besides, computational methods have been reported to improve the quality of co-expression network identification (NetMiner, Yu et al., 2018; PCC-HRR Liesecke et al., 2018). These tools aim toward paving the way to perform co-expression analysis in plant species without limitations.

Leveraging these resources related to network analysis can enhance our understanding in biomass production in different plant species. On one hand, several expression atlas or co-expression resources contains a number of samples from the grass species (*i.e.*, rice, wheat and maize) across stem elongation, thus making possible to identify co-expressed modules associated with stem growth or straw biomass accumulation (Borrill et al., 2016; Kudo et al., 2017; Hoopes et al., 2019; Obayashi et al., 2018). On the other hand, valuable web resources (the AspWood and NorWood database for Populus tremula and Picea abies, respectively) demonstrate the power for generating insights into wood formation and cell wall biosynthesis (Jokipii-Lukkari et al., 2017; Sundell et al., 2017). Moreover, AspWood exemplifies comparative analysis between the coexpression networks from two species, highlighting that conserved coexpression patterns are detected for many processes during wood formation (e.g., cambial growth, secondary cell wall deposition and xylem maturation). In addition, many of the cell wall metabolic regulators identified by coexpression analysis still maintain relatively conserved functions in biomass accumulation in other grasses, such as sorghum (Hennet et al., 2020). To facilitate such comparative analysis between model and non-model species, ATTED and Plant Regulomics have laid foundation for cross-study and cross-species comparisons and retrieving upstream regulators of certain genes of interest (Obayashi et al., 2018; Ran et al., 2020).

While the efforts made in co-expression analyses, three types of challenges remain in: (1) analysis of time-course expression data, (2) inference of gene regulatory networks (GRNs) from the co-expression data, and (3) comparison of co-expression modules between plant species.

First, clustering or co-expression analysis particularly for time-course data emphasizes on capturing the nonstationary time dependence in the data, for which multivariate clustering algorithms or nonlinear regression modelling methods usually perform better than the traditional clustering approaches (Heard et al., 2005). Thus, computational tools such as Smoothing spline clustering (SSClust) (Ma et al., 2006) or tcgsaSeq (Agniel and Hejblum, 2017) have been developed to identify gene clusters from time-course expression data.

Second, new computational approaches have also been available to predict gene regulatory cascade from large-scale RNA-seq data, *e.g.* the nonparametric Bayesian and Markov clustering methods (Penfold et al., 2015a; Penfold et al., 2015b; Desai et al., 2017; Yu et al., 2019). Successful examples have been shown in crops, *i.e.* Harrington et al. (2020) report the GRNs in wheat built with the GENIE3 software. Another group develops the tool HTRgene to specifically extract stress-responsive regulatory network, highlighting the value of GRNs in underpinning particular biological questions (Ahn et al., 2019). Another key to infer GRNs is to identify overrepresented known *cis*-regulatory motifs in the gene promoters that are possibly functional in the regulation of gene expression. Computational search of *cis*-motifs in the promoter region can be readily conducted by using online websites, such as PlantCARE (Lescot et al., 2002), PlantPAN (Chow et al., 2019), or Jaspar (Fornes et al., 2020). Recently, identification of the overrepresented *cis*-motifs has been achieved by the Regulatory Sequence Analysis Tools (RSAT; Nguyen et al., 2018; Ksouri et al., 2021) and its plant-adopted version RSAT-plant (Contreras-Moreira et al., 2016; Ksouri et al., 2021). Lately, resources for visualization and efficient deployment of gene regulatory omics data (ChIP-seq, for instance) have been also available at ChIP-Hub (Fu et al., 2022) and Connec-TF (Brooks et al., 2021), making possible for transferring the TF-target regulatory relationship from the model plants to non-model species.

Last, for the comparison of coexpression networks between species, successful examples have been reported in Brassicaceae (Becker et al., 2017). ATTED-II (Obayashi et al., 2018) is a database hosting 16 co-expression platforms from nine species, allowing the comparison of co-expression modules between the species. In particular, as the resources and tools to move RNA-seq analysis toward regulomics have become mature, the Plant Regulomics database has been built, hosting a huge volume of transcriptomic and epigenomic data sets for six representative species (*i.e.*, Arabidopsis, rice, maize, soybean, tomato and wheat) and enabling the query of upstream regulators of genes (Ran et al., 2020). The Plant Regulomics database sets a nice example for future RNA-seq-centered web interface and analysis direction for other plant species.

### RNA-seq data identify alternative splicing and alternative polyadenylation

While the expression atlas and co-expression analysis are based mainly on gene expression levels, RNA-seq data can also capture structural changes in the transcripts, presenting another layer of regulatory information with biological significance. Two major structural alterations are frequently detected in the transcriptome: (1) Alternative splicing (AS), a phenomenon in which particular exons of a gene may be included or excluded from the processed messenger RNA (mRNA), leading to multiple proteins encoded from a single gene; (2) Alternative polyadenylation (APA), a phenomenon in which a transcript is processed to produce multiple isoforms differing in their untranslated regions (UTRs), in most of the cases, 3’UTRs. Both AS and APA greatly increase the complexity of transcriptome or the repertoire of proteins, and are involved in the molecular, physiological and developmental pathways (Seo et al., 2013; Srivastava et al., 2018). In human, Arabidopsis and maize, respectively, ~95%, 61% and 57% of multi-exonic genes are alternatively spliced, respectively (Pan et al., 2008; Reddy et al., 2013; Wang et al., 2016). In parallel, over 80% and 75% of the genes in human and Arabidopsis respectively can produce multiple mRNA isoforms through APA (Mayr, 2016; Guo et al., 2016). The 3’UTR regions harbor *cis*-acting elements, which regulate various mRNA properties, including RNA stability, transportation, subcellular movement and translation efficiency (Srivastava et al., 2018).

Currently, computational methods for identifying differential AS have been achieved with different quantification schemas, such as those using count-based models (*i.e.*, DEXSeq (Anders et al., 2012), DSGseq (Wang et al., 2013), SpliceCompass (Aschoff et al., 2013), rMATS (Shen et al., 2012), rDiff (Drewe et al., 2013) and RNAprof (Tran et al., 2016)), and those modelling isoform ratios (*i.e.*, Cufflinks and DiffSplice) (Hu et al., 2013). Notably, some new genome assemblies of plants might not have the standard gene annotations as those of human or mouse, and not be readily compatible with some AS quantification tools or need considerable bioinformatic customizations. This issue presents somewhat a technical bar to identify and quantify AS in any plant species, even though identification of differential AS events can be done in major plant species with rMATS and CuffDiff (Liu et al., 2014). Also, new tools for identify intron retention, a particular type of AS frequently seen in plants, has been reported (Mao et al., 2017), enriching the toolbox for AS analysis.

For alternative polyadenylation, user-friendly tools compatible with the genomes of non-model plant species are relatively limited, whereas major efforts have been made to capture 3’UTRs by specific experimental protocols, such as PAT-seq (Harrison et al., 2015), 3’READs (Hoque et al., 2013), and mTAIL-seq (Lim et al., 2016). Only a handful of tools have been reported to identify 3’UTR variations and to calculate differential 3’UTRs using short-read RNA-seq data from plants. The priUTR pipeline detects differential 3’UTR events from Cufflink-derived, genome-guided transcriptome assemblies, discovering the link between 3’UTR and m6A epitranscriptomic modification (Tu and Li, 2020). APAtrap is one of the tools providing flexible and highly efficient APA detection for plant RNA-seq data (Ye et al., 2018). In addition, RNAprof detect both AS and APA events in plant RNA-seq data sets (Tran et al., 2016), while 3D RNA-seq provides three-way differential analysis: differential expression (DE), differential alternative splicing (DAS) and differential transcript usage (DTU) of RNA-seq data (Guo et al., 2021). These recent methods promise the identification of differential AS and APA events as a regular analysis of plant RNA-seq data.

## Discussion and concluding remarks

Many of the short-read, bulk RNA-seq data accumulated today from less-studied plants may be under utilized. Thus, making full use of these data by integrating RNA-seq tools presents an exciting yet challenging prospect. Still, improvements can be made in the following aspects: (1) to integrate with the long-read RNA-seq data; (2) to develop tools or optimize the current pipelines to adapt to complex plant genomes.

PacBio isoform sequencing (Iso-seq) has been the main choice for identifying full-length transcripts. Besides, high-quality full-length isoform sequencing has greatly expanded our understanding in genome annotation, isoform phasing, detection of fusion transcript and alternative splicing and alternative polyadenylation (APA). For example, automated annotation pipelines have been developed to combine the advantages of different annotation methods, including *ab initio* and protein evidence-based prediction and long-read sequencing data (Cook et al., 2018; Tardaguila et al., 2018). However, limited by the medium throughput, Iso-seq-based transcript quantification is far from affordable, especially for the project with a tight budget or a large number of samples. Thus, combining the Iso-seq-derived transcriptome and short-read RNA-seq represents an affordable strategy to both accurately capture a large number of transcripts and to quantify them (**Figure 1**). On another hand, ONT technology has demonstrated its potential in detection of poly(A) tail length and RNA modifications. Therefore, combination of ONT RNA-seq technologies and short-read RNA-seq results will enable novel insights into epitranscriptomic regulation. It is worth to note that while full-length transcriptomes based on the long-read sequencing technologies are apparently advantageous over the short-read RNA-seq in identification of alternative splicing and polyadenylation, tools analyzing short-read sequencing data for these purposes (such as rMATS, rDiff, RNAProf, APAtrap and priUTR) still have their particular niches because short-read RNA-seq are still dominant in the less-studied plant species and are cost affordably for most of the labs, even in high sequencing depth.

In addition, expression quantification may be complicated by other difficulties associated with plant genomes. Polyploid, including both allopolyploid and autopolyploid, are widespread in land plants. Polyploid species are frequent in biomass crops, such as the allopolyploid Miscanthus species (Mitros et al., 2020) and autopolyploid sugarcane species (Zhang et al., 2018). High levels of sequence similarity between the homo-/homoeologous alleles or gene members pose many challenges to the alignment of short reads and subsequent expression quantification. Thus, tools for the RNA-seq analysis of polyploid species or the pipelines tuned for such expression quantification are necessary (Kuo et al., 2018; Paya-Milans et al., 2018), as polyploid species have begun to be assembled recently.

Notably, short-read RNA-seq also has major merits in other plant-related research areas, especially single-cell/single nuclear RNA-seq and meta-transcriptome analysis, owing to the compatibility and cost affordability. Short-read RNA-seq facilitates meta-transcriptome characterization, profiling gene expression in a microbial community and providing a snapshot for functional exploration (Turner et al., 2013; Salazar et al., 2019). In particular, deep RNA-seq can be used to profile the gene expression from both the host and pathogens to obtain insights into plant-microbial interactions (Rudd et al., 2015).

More recently, short-read RNA-seq has been pushed to single-cell resolution due to a series of technological advancements, including robotics, microfluidics and hydrogel droplets (Zhang et al., 2019). In a few years, efforts in single-cell RNA-seq (scRNA-seq) or single-nuclei RNA-seq (snRNA-seq) have expanded from model plants (Arabidopsis, tomato and rice) to non-model species (e.g., maize and poplar), from organ development and cell differentiation to wood formation (Gutzat et al., 2020; Xu et al., 2020; Li et al., 2021; Kajala et al., 2021; Chen et al., 2021a; Wang et al., 2021; Bezrutczyk et al., 2021; Liu et al., 2022). Undoubtedly, single-cell transcriptomics are leading the fore frontier of plant single-cell biology and playing an ever-increasing role in plant research and breeding. Excellent reviews and public database on plant scRNA-seq datasets are available (Shaw et al., 2021; Chen et al., 2021b; Shahan et al., 2021). Due to the differences in several aspects of the wet- and dry-lab parts between the single-cell and bulk RNA-seq experiments, the merits of short-read RNA-seq in single-cell plant biology is beyond the scope of this review and can be found elsewhere (Shaw et al., 2021).

In summary, our work discusses a representative collection of RNA-seq analysis tools covering gene annotation, construction of expression atlas, gene regulation and alternative splicing. We emphasize that the integration of these tools will unleash the power within RNA-seq analysis, uncover the gene regulatory complexity for many less-studied plant species, and, ultimately, promote the functional genomics of these species.

## Author contributions

MT and JiZ developed the conceptual outline and drafted the manuscript. All authors contributed to the article and approved the submitted version.
