# Supplementary material for: Unleashing the power within short-read RNA-seq for plant research: Beyond differential expression analysis and toward regulomics
Source: Front Plant Sci. 2022 Dec 8;13:1038109. doi: 10.3389/fpls.2022.1038109 (PMC9773216; doi:10.3389/fpls.2022.1038109)
Supplement: Supplementary file 1 [file Table_1.docx]

**Supplemental Table S1.** Simplified description of the representative resources and tools for analyzing the short-read RNA-seq data in plants.

| **Name** | **References** | **Description** |
| --- | --- | --- |
| Plant Reactome | Nathani *et al.* 2016 & 2020 | The plant reactome resource(2016) provide annotations of metabolic, transport, developmental and signaling pathways for 62 model, crop and evolutionarily important plant species; the new reactome database(2020) not only provides annotations of metabolic, transport, signaling pathways for plant species, but can also be used for performing online analysis of users’ own RNA-seq data. |
| Strawberry | Liu *et al.* 2017 | Strawberry is a new package for *de novo* assembly of transcriptome with the function of transcript quantification. |
| iDEP | Ge *et al.* 2018 | The authors reported a comprehensive pipeline iDEP that seamlessly connects many R packages and annotation and pthway databases to analyzed RNA-seq data without species limitations. |
| TransFlow | Seoane *et al.* 2018 | TransFlow provides flexible pipelines for multiple *de novo* assembling strategies for RNA-seq data, with the capability to integrate Roche/454 sequencing data. |
| MorphDB | Zwaenepoel *et al.* 2018 | The authors provide an online database, MorphDB, which integrate multiple annotation resources for identification of missing gene in metabolic mathways in plants. They also provide the toolkit for local prediction of metabolic annotation for novel data sets, MORPH bulk. |
| PISO | Feng *et al.*2019 | The plant isoform sequencing database (PISO) provided millions of high-quality transcript isoforms from 19 plant species with a flexible user interface and conventional tools. |
| MapMan 4/ Mercator4 | Schwacke *et al.*2019 | The authors presented a redesigned and significantly enhanced annotation database and annotation software, MapMan4. |
| PlantCircBase | Chu *et al.*2018 | The paper reports an update of plant circular RNA database, PlantcircBase 3.0, including the identified circRNAs from 12 plant species with less false positive predictions. |
| Gramene | Tello-Ruiz *et al.*2018 | This paper described the integrative knowledgebase for crop plants, Gramene, which includes 44 reference genomes of crops, the annotation of gene families and plant reactome annotations. It also hosts the expression atlas of several crops. Its pathway annotation has been further enhanced by the curated rice pathways and gene orthology between the species. |
| **Name** | **References** | **Description** |
| LeGOO | Carrere *et al.* 2020 | This paper described a knowledgebase of the model legume species, Medicago truncatula (namely LeGOO). The knowledgebase hosts functional relations retrieved from 298 publications, the genome broweser implemented with the Mt5.0 reference genome, and the associated functional annotations. Thus, this database will be useful for the legume community to better exploit the wealth of data from this model species. |
| ZEAMAP | Gui *et al.* 2020 | This paper described a comprehensive website (ZEAMAP) for functional and comparative genomics of maize. The ZEAMAP websites includes multiple reference genomes, comparative transcriptomes, chromatin data, and high-quality variants, phenotypes, metabolomics and even genetic maps. |
| BarleyNet | Lee *et al.* 2020 | This paper described a repository of co-functional network of barley and its companion webserver (BarleyNet). The barleyNet co-functional networks host 2385 expression profiles (1780 by microarray data and 650 by RNA-seq data). The authors further desmontrated the usefulness of these network analysis tools in the study of stress responses. |
| SAT-Assembler | Zhang *et al.* 2014 | SAT- assembler uses RNA-seq data to perform gene family-specific assembly with high homology and lower chimera rate. |
| BinPacker | Liu *et al.* 2016 | BinPacker is a new software for *de novo* assembly of transcriptome with higher accuracy, faster and requires less memory. |
| Rascaf | Song *et al.* 2016 | The program Rascaf was developed to leverage the splice junction-spanning read of RNA-seq to detect new contig connections, facilitating contig improvement and thus genome assembly. |
| IGB | Freese *et al.* 2016 | The paper reports updates of the Integrated Genome Browser (IGB), a powerful tool to integrate multiple omic data sets. |
| eFP-Seq Browser | Sullivan *et al.* 2019 | This paper described a website links two powerful tools for analyzing large RNA-seq datasets, the electronic fluorescent pictographic (eFP) and Integrated Genome Browser (IGB) to enhance the visualization and exploration of RNA-seq data sets for plant biologists. |
| RNAprof | Tran *et al.*2016 | The authors developed a program (namely RNA profile analysis, RNAprof) to detect differential RNA processing events (i.e., alternative splicing and variation in polyadenylation) and demonstrated its validity in the RNA-seq data sets from both animal and plant species. |
|  |  |  |
| **Name** | **References** | **Description** |
| Apatrap | Ye *et al.*2018 | The authors developed a software (APAtrap) specifically detect novel 3’UTRs and extensions of 3’UTRs using RNA-seq data. They showed the high efficacy and flexibility of APAtrap by extensive comparison between similar programs. |
| priUTR | Tu *et al.*2020 | The authors report a pipeline priUTR that identifies differential 3’UTR events and is compatible with classic pipelines for RNA-seq differential expression analysis (the Tophat-Cufflinks pipeline, or the HISAT-StringTie pipeline). With the aid of the priUTR, the authors highlighted the association of alternative 3’UTRs with m6A modification. |
| 3D RNA-Seq | Guo *et al.* 2021 | The authors developed “3D RNA-seq”, a R shiny App which provides three-way differential analysis: Differential Expression (DE), Differential Alternative Splicing (DAS) and Differential Transcript Usage (DTU) of RNA-seq data. The author further demonstrated its compatibility with Arabidopsis and mouse RNA-seq data. |
| TEtranscripts | Jin *et al.* 2015 | The program Tetranscripts was developed to recover transposable elements derived transcripts and perform analysis, which exists in a large proportion in many plant species. |
| expVIP | Borrill *et al.* 2016 | The authors developed a flexibile RNA-seq pipeline and analyzed 16 wheat RNA-seq studies (including 418 individual samples). This wheat expression atlas has been accessed and easily visualized in the expVIP database. |
| OryzaExpress | Kudo *et al.* 2016 | The authors reported a web database OryzaExpress in which gene expression atlas of rice determined by microarray and RNA-seq have been deposited and integrated. |
| BAR | Waese *et al.*2016 | The Bio-Analytic Resource (BAR) website provides online integration of large omic datasets for 15 different plant species. |
| DPMIND | Fei *et al.*2018 | miRNAs are an important group of regulatory components encoded in the plant genomes; however, miRNA and their target genes remain to be undertstood in many species. The degradome-based plant miRNA–target interaction and network database (DPMIND) has been built on 69 degradome sequencing data sets from 10 plant species, providing very useful knowledge to expand our understanding of plant RNA-seq data. |
| PEATmoss | Fernandez-Pizo *et al.*2019 | The Physcomitrella Expression Atlas Tool (PEATmoss) collected and analyzed 109 RNA-seq experiments from nine data sets, together with P. patens annotations and other useful tools. |
| **Name** | **References** | **Description** |
| ASmir | Wang *et al.*2019 | The alternatively spliced linear and circRNAs (ASmiR) database takes advantages of existing Iso-seq and miRNA sequencing data and compiled the interplay between alternative splicing of linear and circular RNAs and miRNA-targeting. This database provides additional information for understanding AS-mediated mRNA-miRNA interactions. |
| Soybean Expression Atlas | Machado *et al.*2020 | The soybean expression atlas has collected and analyzed 1298 publicly available RNA-seq samples with identification of house-keeping and tissue-specific genes and transcript-level quantification. |
| Grape-RNA | Wang *et al.* 2020 | This paper described a database of grape RNA-seq data sets, including 1529 RNA-seq samples and 112 miRNA sequencing samples together with weel-documented metadata. |
| CORNET | Van Bel *et al.*2016 | The authors reported the website (CORrelation NETwork, CORNET 3.0) allowing the calculation of correlation-based co-expression networks in plant species. The protocol of this website also indicates how to avoid common pitfalls and how to avoid overinterpretation of the results. |
| NaDH | Brockmoller *et al.*2017 | The Nicotiana attenuata Data Hub (NaDH) is a centralized data hub for the Nicotiana species, hosting hundreds of microarray data and 20 RNA-seq experiments with enhanced visualization and co-expression functions. |
| NorWood | Jokipii-Lukkari *et al.* 2017 | The authors established the NorWood database, which hosts a large volume of high-spatial-resolution section series RNA-seq data of Picea abies. The database also provides co-expression networks with a focus on transcriptional regulation associated with cell wall metabolism. |
| AspWood | Sundell *et al.*2017 | The authors established the AspWood database, which hosts a large volume of high-spatial-resolution section series RNA-seq data of Populus tremula. |
| RED | Xia *et al.* 2017 | The authors introduced the Rice Expression Database (RED) which hosts 284 high-quality RNA-seq datasets of rice and enables the inquiry of house-keeping and tissue-specific genes as well as co-expression networks in rice. |
| EXPath | Zheng *et al.*2017 | The EXPath Tool is an integrative website containing multiple functions, including differential expression analysis, co-expression network analysis, GO enrichment and comparative network analysis between data sets. |
| **Name** | **References** | **Description** |
| TomExpress | Zouine *et al.* 2017 | The author builds a comprehensive expression atlas of tomato containing 349 RNA-seq samples corresponding to 222 conditions. The database not only provides a user-friendly visualization of the expression atlas but also includes visulization of co-expression networks and other useful functions. |
| Maize eFP Brower | Hoopes *et al.*2018 | The authors build one of the largest expression atlas of maize consisting 79 tissues and various stress treatments. Co-expression network analysis shed light on molecular mechanisms of stress tolerance and the connections between presence-absence variations (PAVs) and stress-inducible genes. |
| ATTED | Obayashi *et al.*2018 | The ATTED database provides 16 co-expression platforms for nine plant species with robust statistical information. |
| MCENet | Tian *et al.*2018 | The authors built a database (called the maize conditional co-expression network, MCENet) hosting 701 maize transcriptomic data and 108 epigenomic data. In addition, the database enables co-expression network analysis and functional toolkit for further data mining of the co-expression modules. |
| AppleMDO | Da *et al.*2019 | This paper described a comprehensive omics database of apple (Malus domestica Borkh.) (namely AppleMDO), which includes 112 RNA-seq datasets and 20 epigenomic datasets (ChIP-seq, DNase-seq and DNA methylation data). The website also provides global networks and conditional networks for the apple omics data. |
| Melonet-DB | Yano *et al.*2018 | This paper described a database of Melon (Cucumis melo L.) (Melonet-DB), including an expression atlas of Melon and weighted gene co-expression network. The authors demonstrated the ablity of Melonet-DB to promote functional genomic studies. |
| TPIA | Xia *et al.* 2019 | This paper described a comprehensive knowledgebase of tea plant (Tea Plant Information Archive, TPIA), which includes abundant weel-organized transcriptomes, curated annotations (especially those genes particularly important for the tea quality) and a variety of versatile useful analytical tools. |
| Plant Regulomics | Ran *et al.* 2020 | This paper described a comprehensive database containing 19,925 transcriptomic and epigenomic data sets and diverse functional evidence of six plant species (Arabidopsis, rice, maize, soybean, tomato and wheat) along with the orthologous genes from 56 whole-genome sequences species. |
| CSI | Penfold *et al.* 2015a & 2015b | The causal structural identification (CSI) package employes a nonparametric Bayesian approach to infer gene regulatory networks (GRNs) with graphical user interface (GUI), allowing implementation in MATLAB. |
|  |  |  |
| **Name** | **References** | **Description** |
| RSAT-Plants | Contreras-Moreira *et al.* 2016 | The authors report the plant-dedicated website of Regulatory Sequence Analysis Tools (RSAT-Plants) and a step-by-step protocol for motif discovery and the identification of TF-target regulation. |
| tcgsaseq | Agniel *et al.* 2017 | The tcgsaseq R package offers the identification of gene sets with different expression patterns in time-series RNA-seq data with good statistical properties. |
| SeqEnrich | Becker *et al.*2017 | The software is designed to produce co-expression networks in Arabidopsis and Brassica species and predict transcription factor-DNA interactions with integration of TF knowledge in the species. |
| ExRANGES | Desai *et al.* 2017 | The R package ExRANGES combines expression levels and the rate of changes to improve the accuracy of computational inference of transcriptional regulatory network (TRNs). |
| LSTrAP | Proost *et al.*2017 | LSTrAP (Large-Scale Transcriptome Analysis Pipeline) is single, efficient and flexible workflow for plant RNA-seq analysis, from fastq data processing to co-expression network analysis, allowing hundreds of RNA-seq samples being processed efficiently. |
| RSAT | Nguyen *et al.*2018 | The paper reports the update of Regulatory Sequence Analysis Tools (RSAT) supporting the discovery of motifs in over 10,000 genomes from all kingdoms. |
| NetMiner | Yu *et al.* 2018 | By integrating three frequently used co-expression network inference methods, the NetMiner program provides a reliable approach to calculate co-expression networks. |
| ExpressWeb | Savelli *et al.* 2019 | The ExpressWeb allows user-friendly gene clustering and co-expression network identification and integration of transcriptomic and proteomic datasets. |
| HTRgene | Ahn *et al.* 2019 | The authors developed a specific algorithum HTRgene with high performance in identifying stress responsive gene networks. |
| Compare Transcriptome Analysis | Lee *et al.* 2019 | The authors described a new approach to compare co-expression networks between species, particularly the time series transcriptomme data. |
| **Name** | **References** | **Description** |
| JASPAR | Fornes *et al.* 2020 | The Jaspar database hosts curated, non-redundant transcription factor-binding profiles from species in six taxnomic groups. The database also comes together with multiple tools for gene regulation analysis. |
| GENIE3 | Harrington *et al.* 2020 | The authors retrieved multiple independent and publicly available RNA-seq datasets and constructed wheat GRNs by GENIE3 method, and validated the GRNs by functional study of the NAM-A1 gene. |
| LSTrAP-Cloud | Tan *et al.* 2020 | The authors implemented the classic kallisto pipeline for RNA-seq analysis and co-expression network analysis in cloud, namely LSTrAP-Cloud |
| RSAT | Ksouri *et al.*2021 | The regulatory sequence analysis tools (RSAT) is a classic software for identifying regulatory sequences in the genome. To facilitate its utilization in plant species, the authors developed RSAT-plants and implemented in an easy manner, RSAT-docker. They demonstrated the power of RSAT-plants in mining transcriptome data and regulaotry networks. |
